# 1124. Case Study. Improved Cosmetic Appearance of a Modified Cultured Epidermal Autograft Compared to Traditional Skin Autograft

**DOI:** 10.1093/jbcr/irag033.527

**Published:** 2026-04-06

**Authors:** Wayne George Kleintjes, Tarryn Kay Prinsloo

**Affiliations:** Sheik Khalifa Hospital Fujairah, Cape Town, Western Cape; Cape Peninsula University of Technology, Cape Town, Western Cape

## Abstract

**Patient Presentation (age range, injury details, relevant history):**

A 54-year-old male with deep partial-thickness flame burns on both forearms.

**Clinical Challenges:**

Cultured epidermal autografts (CEA) are limited by logistical challenges, high costs, and handling difficulties, which has restricted their use mainly to life-saving treatment of severe burns. Reports on CEA in cosmetic applications remain scarce, despite evidence suggesting superior long-term aesthetic outcomes. In a resource-limited setting, a modified, low-cost technique was developed to treat burn patients with poor survival outcomes. We hypothesized that this modified technique could provide improved cosmetic results compared with traditional autografting, offering a viable option for patients prioritizing wound appearance.

**Management Approach:**

The modified technique involved immersing epidermal fragments, separated from a 3 × 2 cm full-thickness skin biopsy, in trypsin for 2 hours. The resultant keratinocytes were seeded onto routinely used dressings and incubated in pediatric incubators at 37°C for 2 weeks until confluence. Culture supplementation included daily application of fresh autologous plasma and hydrogel every 3–4 days. Xenografts were applied during the culture period, removed thereafter, and the CEA-containing dressing transplanted directly onto the debrided wounds of the right forearm. Traditional split-thickness autografts were applied to the contralateral forearm. Both forearms sustained deep partial-thickness flame burns. Healing and appearance were observationally assessed in terms of pigmentation, texture, and pliability at 2 weeks, 1 month, and 1 year.

**Outcomes:**

At 2 weeks post-transplant, CEA demonstrated less scabbing than the autograft. By 1 month, healing outcomes appeared comparable between groups. However, with long-term healing at 1 year, the CEA site demonstrated greater pliability, secondary hair growth, and a smoother overall appearance compared with the traditional autograft.

**Lessons Learned:**

The favorable long-term cosmetic outcomes associated with the modified CEA technique are consistent with previous limited reports and support its potential for broader cosmetic applications. Further research is needed to validate these findings across diverse populations, burn depths, and comorbidities, as well as to compare outcomes with commercially available CEAs.

**Applicability to Practice:**

This case demonstrates that a low-cost, modified CEA technique can provide superior cosmetic outcomes compared with traditional autografts, even in resource-limited settings. If validated in larger studies, this approach could expand reconstructive options for burn patients where cosmesis is a primary concern, offering an affordable alternative to commercial CEAs and potentially improving long-term quality of life.

